# LPDA: A new classification method based on linear programming

**DOI:** 10.1371/journal.pone.0270403

**Published:** 2022-07-07

**Authors:** María J. Nueda, Carmen Gandía, Mariola D. Molina

**Affiliations:** Mathematics Department, University of Alicante, Alicante, Spain; Dartmouth College Geisel School of Medicine, UNITED STATES

## Abstract

The search of separation hyperplanes is an efficient way to find rules with classification purposes. This paper presents an alternative mathematical programming formulation to existing methods to find a discriminant hyperplane. The hyperplane *H* is found by minimizing the sum of all the distances to the area assigned to the group each individual belongs to. It results in a convex optimization problem for which we find an equivalent linear programming problem. We demonstrate that *H* exists when the centroids of the two groups are not equal. The method is effective dealing with low and high dimensional data where reduction of the dimension is proposed to avoid overfitting problems. We show the performance of this approach with different data sets and comparisons with other classifications methods. The method is called *LPDA* and it is implemented in a R package available in https://github.com/mjnueda/lpda.

## Introduction

One of the main goals in many recent data analysis projects is the classification of samples or individuals into predefined groups, according to the characteristics available. Several approaches have been proposed to deal with this problem. Statistical methods, usually are based in the evaluation of a scoring function that needs distributional assumptions as Fisher Linear Discriminant Analysis (*LDA*) [1, 2] or Logistic Regression [3]. The high number of variables and the diverse type of distributional assumptions are challenging topics that researchers try to solve with non distributional approaches. Mathematical programming is a natural way of dealing with the classification problem regardless of distributional assumptions. In this sense, linear programming based methods look for a linear function that separates the classes avoiding parameters estimations. Support Vector Machine (*SVM*) [4, 5] is the most popular classification method based in hyperplanes, that can be extended to nonlinear separating functions, as polynomial or radial kernel. In [6] we find a discussion of mathematical optimization techniques proposed for SVM and [7] reviews and compares supervised classification methods related to optimization. These publications and other as [8] demonstrate the exinting interest of addressing the classification problem through mathematical programming. We can also mention the Machine Learning approach, where we find alternative methods as Decision Trees, CART or Random Forest, [9, 10] and Neural Networks approach [11]. This approach tries to find a stepwise rule that combines the best ranking variables in a training set also ignoring distributional assumptions.

All these approaches could be considered complementary rather than competitive. Machine learning approaches are useful in classification when dealing with high dimensional data sets, but for interpreting variable influence it is preferable Logistic Regression or *LDA*. *SVM* is an effective method in different situations. When dealing with small dimension the flexibility of the separating function can help to find a perfect separation, however with high dimensional data over-fitted problems can emerge and, as mentioned in [12], there is not need of additional flexibility that give this models, being the linear function a good option.

In this work we propose an efficient alternative to the available classification methods in R without distributional assumptions. We formulate an optimization problem to find a discriminating hyperplane between two data sets that can be useful to classify new individuals. The method has been extended also to the case with more than two groups making paiwise comparisons. In addition, to avoid overfiting problems due to noisy data or high dimensional data sets, we consider Principal Components Analysis (PCA) to focus on the main sources of variation avoiding the noise. The method has been implemented in a R package named **lpda** available in github.

The paper is structured as follows. In the following section, the optimization problem is proposed on the basis of the general two-group classification approach and the PCA solution is presented. Then, it is described the evaluation strategy of the new technique: data and other approaches against which it is intended to be compared. In the Results section this evaluation is showed and finally, conclusions are presented in the last section.

## Linear programming discriminant analysis method

The purpose of this section is to describe the problem we want to solve and to build the linear problem which will allow us to find the solutions. First, we present the approach for the case of two data sets and subsequently extend it to the case with more than two sets. Finally, we propose a strategy to avoid overfitting in data sets with more variables than individuals.

### Model definition for two data sets

Let $X=\{x_{1},...,x_{n_{1}}\}$ and $Y=\{y_{1},...,y_{n_{2}}\}$ two sets whose elements are in $R^{p}$, and $m^{t}=\left(m_{1},m_{2},...,m_{n_{1}}\right)$ and $w^{t}=\left(w_{1},w_{2},...,w_{n_{2}}\right)$ the vectors whose components are the weights of the elements of **X** and **Y** respectively, positive and such that $\sum_{i=1}^{n_{1}}m_{i}=\sum_{j=1}^{n_{2}}w_{j}=1$.

Weights can be assigned depending on the importance of the individual in the sample. This could be of interest if the individuals are collectives; for example: cities or universities; that can be weighted by their size. If all the individuals are equally important, weights must be *m*_*i*_ = 1/*n*_1_∀*i* and *w*_*j*_ = 1/*n*_2_∀*j*.

**Definition 1**. *A hyperplane H in* $R^{p}$ *is an* (*p*-1)-*affine set and can be represented as* $H=\{x\in R^{p}|a^{t}x=b\}$, *where* b∈R *and* $a\in R^{p}$, *a* ≠ 0_*p*_, *and they are unique up to a common non-zero multiple*.

**Fig 1:**
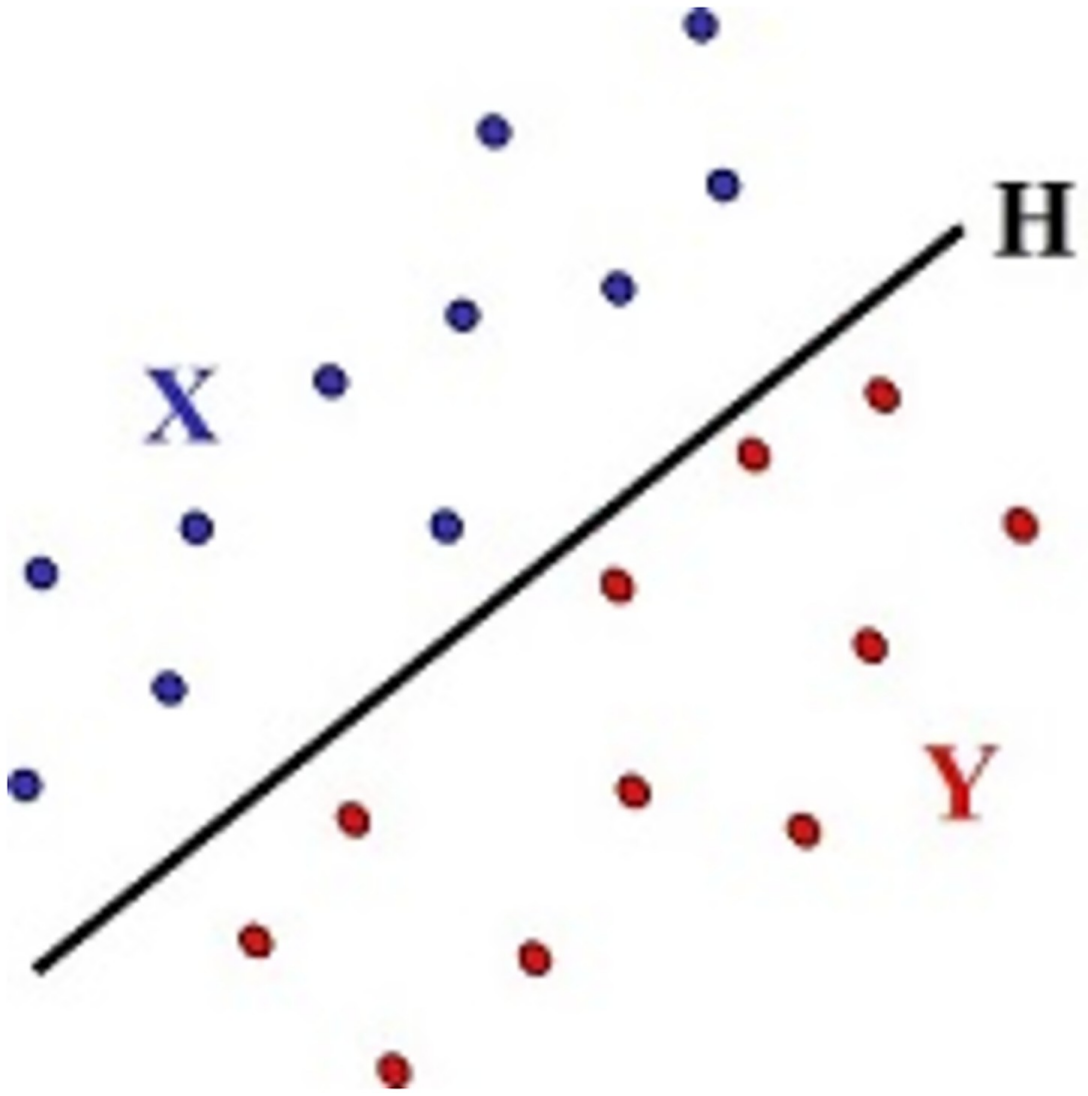
The objective is finding *H* that separates X from Y.

Initially, we look for a hyperplane *H* that strictly separates **X** from **Y** (Fig 1). If such hyperplane does not exist, we focus on a hyperplane that minimizes a measure of the deviation of this goal, called *separation error*.

**Proposition 1**. ***X*** *and* ***Y*** *are strictly separable if and only if there exists a hyperplane* $H=\{x\in R^{n}|a^{t}x=b\}$ *such that the following system*:

$$\begin{array}{c} \sigma =\{\begin{array}{c} a^{t}x_{i}\geq b+1,\;i=1,\ldots ,n_{1}\\ a^{t}y_{j}\leq b-1,\;j=1,\ldots ,n_{2} \end{array} \end{array}$$ (1)

*is consistent. Such hyperplane is named separator hyperplane*.

Proof.

If **X** and **Y** can be strictly separated, there exists $c\in R^{p}$, *c* ≠ 0_*p*_ and d∈R such that:

$$\begin{array}{c} \begin{array}{c} c^{t}x_{i}>d,\;i=1,...,n_{1}\\ c^{t}y_{j}<d,\;j=1,...,n_{2} \end{array} \end{array}$$ (2)

Let *ε*_*i*_ and *δ*_*j*_ the slacks of each constraint in (2):

$$\begin{array}{c} \varepsilon_{i}=c^{t}x_{i}-d>0,\;i=1,...,n_{1}\\ \delta_{j}=d-c^{t}y_{j}>0,\;j=1,...,n_{2} \end{array}$$

and *η* ≔ min{*ε*_*i*_, *δ*_*j*_}. We can define

$$\begin{array}{c} \left(a^{t},b\right)=\eta^{-1}\left(c^{t},d\right), \end{array}$$ (3)

where $a\in R^{p}$, *a* ≠ 0_*p*_ and b∈R.

Multiplying both sides of (3) by ${\left(x_{i}^{t},-1\right)}^{t}$ we have:

$$a^{t}x_{i}-b=\eta^{-1}\left(c^{t}x_{i}-d\right)=\eta^{-1}\varepsilon_{i}\geq 1,i=1,\ldots ,n_{1}$$

Similarly, multiplying (3) by ${\left(y_{j}^{t},-1\right)}^{t}$ we have:

$$a^{t}y_{j}-b=\eta^{-1}\left(c^{t}y_{j}-d\right)=-\eta^{-1}\delta_{j}\leq -1,j=1,\ldots ,n_{2}$$

Therefore, the pair (*a*, *b*) is a solution of the system (1).

Conversely, if the system (1) has a solution (*a*, *b*), then $H=\{x\in R^{p}|a^{t}x=b\}$ verifies

$$\left\{ \begin{array}{c} a^{t}x_{i}\geq b+1>b,\;i=1,\ldots ,n_{1}\\ a^{t}y_{j}\leq b-1<b,\;j=1,\ldots ,n_{2} \end{array}\right.$$

Moreover, *a* ≠ 0_*p*_ (otherwise, *b* + 1 ≤ 0 ≤ −1). Hence, *H* is a hyperplane separating strictly **X** and **Y**.

**Fig 2:**
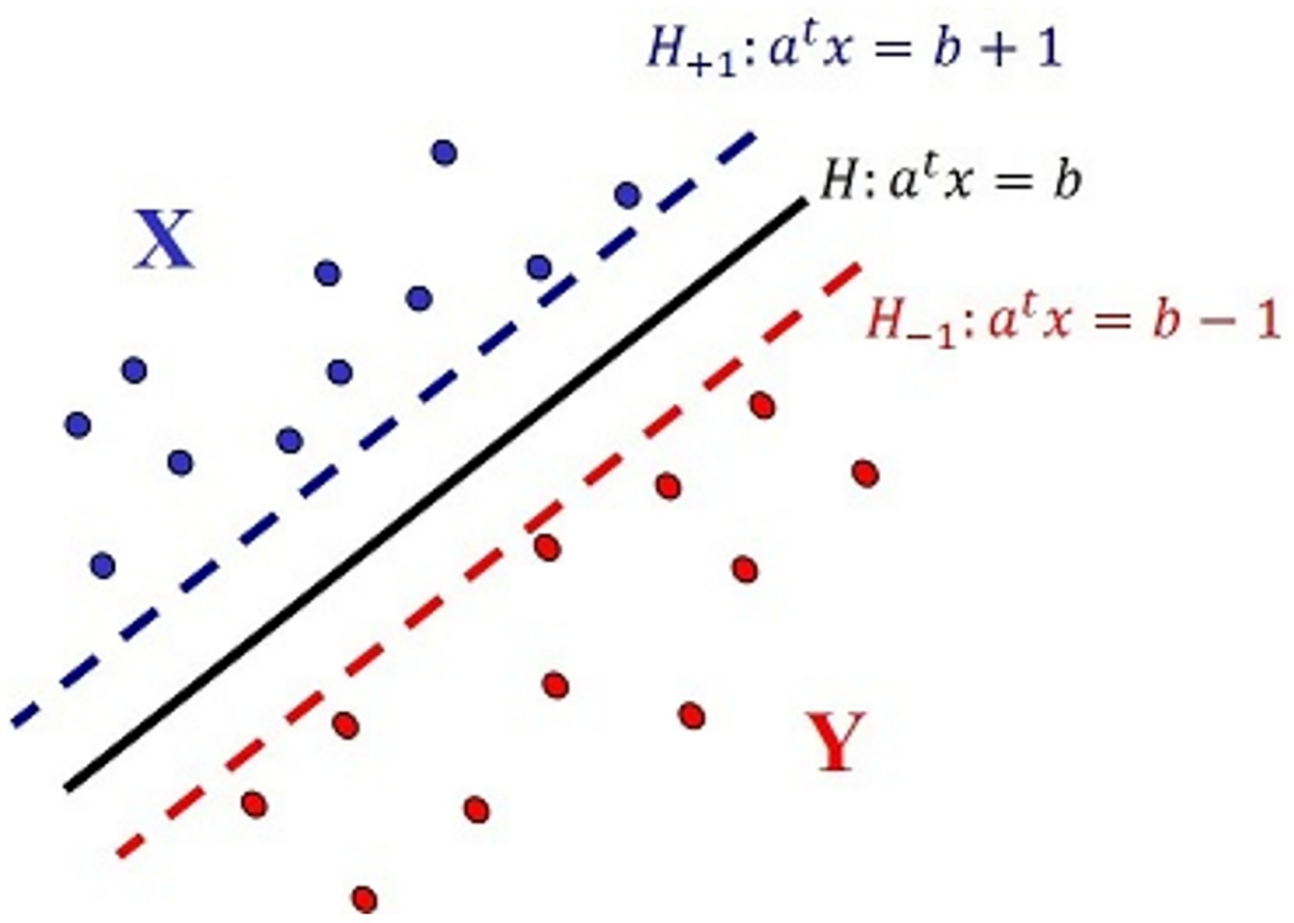
Situation of X and Y related to *H*, *H*_+1_ and *H*_−1_.

Such hyperplane will be referred to as a *separator hyperplane*. This proposition leads us to locate sets **X** and **Y** as it is showed in Fig 2, regarding the hyperplanes

$$H=\{x\in R^{p}|a^{t}x=b\},$$

$$H_{+1}=\left\{x\in R^{p}|a^{t}x=b+1\right\}$$

and

$$H_{-1}=\left\{x\in R^{p}|a^{t}x=b-1\right\}.$$

On the other hand, if (1) is an inconsistient system, there exists some *i* ∈ {1, …, *n*_1_} for which *b* + 1 − *a*^*t*^ *x*_*i*_ > 0 or *j* ∈ {1, …, *n*_2_} for which *a*^*t*^ *y*_*j*_ − *b* + 1 > 0. Therefore, we can take the following values as error measure of each element:

$$\begin{array}{c} \text{max}\left\{b+1-a^{t}x_{i},0\right\},\;i=1,\ldots ,n_{1}\\ \text{max}\left\{a^{t}y_{j}-b+1,0\right\},\;j=1,\ldots ,n_{2}. \end{array}$$

Adding all these measures weighted by *m*_*i*_ and *w*_*j*_, respectively, we obtain the function *f*, called *separation error function*:

$$\begin{array}{c} f\left(a,b\right):=\sum_{i=1}^{n_{1}}m_{i}\;\text{max}\left\{b+1-a^{t}x_{i},0\right\}+\sum_{j=1}^{n_{2}}w_{j}\;\text{max}\left\{-b+1+a^{t}y_{j},0\right\}. \end{array}$$ (4)

The separation error function is a non-negative, convex and non-differenciable function and the aim is to solve the problem

$$(P_{1})\text{min}\;f\left(a,b\right).$$

**Proposition 2**. *σ is consistent if and only if v*(*P*_1_) = 0. *In such case, any optimal solution of P_1_ defines a separator hyperplane*.

Proof.

If **X** and **Y** can be strictly separated, there exists $\left(\bar{a},\bar{b}\right)$ solution of *σ*. So, $f(\bar{a},\bar{b})=0$ and $\left(\bar{a},\bar{b}\right)$ is an optimal solution of (*P*_1_).

Conversely, if $\left(\bar{a},\bar{b}\right)$ is an optimal solution of (*P*_1_), each term in *f* will be equal to zero and by Proposition 1, **X** and **Y** can be separated strictly. Moreover, $\bar{a}\neq 0_{p}$ because $f(0_{p},\bar{b})=2$ and it can not be an optimal solution of (*P*_1_).

So, **X** and **Y** can be strictly separated if and only if *v*(*P*_1_) = 0. But, in any case, the objective is translated in finding the solution $\left(\bar{a},\bar{b}\right)$ to the problem (*P*_1_). We approach this task through a linear problem equivalent to (*P*_1_), whose optimal solutions will define our discriminant hyperplane, that is, the hyperplane that minimizes the separation error function.

**Proposition 3**. (*P*_1_) *is equivalent to the problem*

$$\begin{array}{c} \begin{array}{ccc} \left(P\right) & Min\;\sum_{i=1}^{n_{1}}m_{i}u_{i}+\sum_{j=1}^{n_{2}}w_{j}v_{j} & \\ & s.t. & \\ & \;u_{i}\geq -a^{t}x_{i}+b+1, & i=1,\ldots ,n_{1}\\ & \;u_{i}\geq 0 & i=1,\ldots ,n_{1}\\ & \;v_{j}\geq a^{t}y_{j}-b+1, & j=1,\ldots ,n_{2}\\ & \;v_{j}\geq 0 & j=1,\ldots ,n_{2} \end{array} \end{array}$$

*where the objective is finding* (*a*, *b*), *that define the hyperplane, with the support of the variables u*_*i*_ *and v*_*j*_ *that identify potential errors to be minimized*.

Proof.

Since each of the functions to maximize in each operand in (4) is convex, we have

$$\text{max}\left\{b+1-a^{t}x_{i},0\right\}=\text{min}\left\{u_{i}\in R_{+}|u_{i}\geq b+1-a^{t}x_{i}\right\}$$

and

$$\text{max}\left\{a^{t}y_{j}-b+1,0\right\}=\text{min}\left\{v_{j}\in R_{+}|v_{j}\geq a^{t}y_{j}-b+1\right\}$$

It allows us to reformulate our initial problem as the equivalent problem (*P*) in the following sense [13]:

1. If $\left(\bar{a},\bar{b}\right)$ is an optimal solution of (*P*_1_), then taking
  $${\bar{u}}_{i}=\text{max}\left\{-{\bar{a}}^{t}x_{i}+\bar{b}+1,0\right\}\text{,}\;i=1,\ldots ,n_{1}$$
  and
  $${\bar{v}}_{j}=\text{max}\left\{-\bar{b}+1+{\bar{a}}^{t}y_{j},0\right\}\text{,}\;j=1,\ldots ,n_{2},$$
  then $\left(\bar{a},\bar{b},\bar{u},\bar{v}\right)$ is an optimal solution of (*P*).
2. If $\left(\bar{a},\bar{b},\bar{u},\bar{v}\right)$ is an optimal solution of (*P*), then ${\bar{u}}_{i}=\text{max}\left\{\bar{b}+1-{\bar{a}}^{t}x_{i},0\right\},$ for *i* = 1, …, *n*_1_ and ${\bar{v}}_{j}=\text{max}\left\{-\bar{b}+1+{\bar{a}}^{t}y_{j},0\right\}\text{,}\;j=1,\ldots ,n_{2},$ and $\left(\bar{a},\bar{b}\right)$ is an optimal solution of (*P*_1_).

(*P*) is a solvable problem and every optimal solution will provide a discriminant hyperplane that minimizes the separation error as long as *a* ≠ 0_*p*_. We can state that if *v*(*P*) = 0, this situation is guaranteed by Proposition 2 but, if *v*(*P*) > 0, we need to add a very weak condition on the data sets (in the sense that it will usually be verified), what we will prove in the following proposition. Let us remember that in a linear problem, a necessary and sufficient condition for $\bar{x}$ to be an optimal solution is that the objective vector can be written as a non-negative linear combination of the active constraints on $\bar{x}$.

**Proposition 4** *If v*(*P*) > 0 *and* $\bar{x}\neq \bar{y}$, *with* $\bar{x}=\sum_{i=1}^{n_{1}}m_{i}x_{i}$ *and* $\bar{y}=\sum_{j=1}^{n_{2}}w_{j}y_{j}$ *there exist an optimal solution of* (*P*) *that gives a discriminant hyperplane*.

Proof.

Let us suppose that $\left(\bar{a},\bar{b},\bar{u},\bar{v}\right)$ is an optimal solution of (*P*) with $\bar{a}=0_{p}$. Then,

$$\begin{array}{c} u_{i}\geq b+1,\;\text{for}\;\text{all}\;i=1,\ldots ,n_{1} \end{array}$$ (5)

$$\begin{array}{c} v_{j}\geq 1-b,\;\text{for}\;\text{all}\;j=1,\ldots ,n_{2} \end{array}$$ (6)

and all of them will be active (otherwise, the solution is no an optimal solution). So we can consider $\bar{u}=\left(\bar{b}+1\right)1_{n_{1}}$ and $\bar{v}=\left(1-\bar{b}\right)1_{n_{2}}$, where $1_{n_{1}}$ and $1_{n_{2}}$ are vectors with all its elements equal to 1 in $R^{n_{1}}$ and $R^{n_{2}}$, respectively. Then,

$$v\left(P\right)=\sum_{i=1}^{n_{1}}m_{i}\left(\bar{b}+1\right)+\sum_{j=1}^{n_{2}}w_{j}\left(1-\bar{b}\right)=2.$$

We will distinguish the different values of *b* in order to determine the active constraints in each case and apply the condition that characterizes the optimality.

(a) |*b*| ≠ 1. Now, the unique active constraints are (5) and (6) and hence,
  $$\begin{array}{c} (\begin{array}{c} 0_{p}\\ 0\\ m\\ w \end{array})=\sum_{i=1}^{n_{1}}\lambda_{i}(\begin{array}{c} x_{i}\\ -1\\ I_{n_{1}}^{i}\\ 0_{n_{2}} \end{array})+\sum_{j=1}^{n_{2}}\mu_{j}(\begin{array}{c} -y_{j}\\ 1\\ 0_{n_{1}}\\ I_{n_{2}}^{j} \end{array}), \end{array}$$ (7)
  where λ_*i*_ and *μ*_*j*_ belong to $R_{+}$, for all *i* = 1, …, *n*_1_ and *j* = 1, …, *n*_2_; $I_{n_{1}}^{i}$ and $I_{n_{2}}^{j}$ are the *i*th and *j*th vectors of the canonical basis in $R^{n_{1}}$ and $R^{n_{2}}$, respectively. Then,
  $$\lambda_{i}=m_{i},\text{for}\;\text{all}\;i=1,2...,n_{1},$$
  $$\mu_{j}=w_{j},\text{for}\;\text{all}\;j=1,2...,n_{2}$$
  whereas
  $$0_{p}=\sum_{i=1}^{n_{1}}\lambda_{i}x_{i}+\sum_{j=1}^{n_{2}}\mu_{j}\left(-y_{j}\right)=\bar{x}-\bar{y}.$$
(b) *b* = 1. Now, in addition to (5) and (6), constraints
  $$v_{j}\geq 0,j=1,\ldots ,n_{2}$$
  are active too. Hence,
  $$\begin{array}{c} (\begin{array}{c} 0_{p}\\ 0\\ m\\ w \end{array})=\sum_{i=1}^{n_{1}}\lambda_{i}(\begin{array}{c} x_{i}\\ -1\\ I_{n_{1}}^{i}\\ 0_{n_{2}} \end{array})+\sum_{j=1}^{n_{2}}\mu_{j}(\begin{array}{c} -y_{j}\\ 1\\ 0_{n_{1}}\\ I_{n_{2}}^{j} \end{array})+\sum_{j=1}^{n_{2}}\delta_{j}(\begin{array}{c} 0_{n}\\ 0\\ 0_{n_{1}}\\ I_{n_{2}}^{j} \end{array}), \end{array}$$ (8)
  where λ_*i*_, *μ*_*j*_ and *δ*_*j*_ belong to $R_{+}$, for all *i* = 1, …, *n*_1_ and *j* = 1, …, *n*_2_. Hence,
  $$\lambda_{i}=m_{i},\text{for}\;\text{all}\;i=1,2...,n_{1},$$
  $$\mu_{j}+\delta_{j}=w_{j},\text{for}\;\text{all}\;j=1,2...,n_{2},$$
  whereas
  $$0=\sum_{i=1}^{n_{1}}\lambda_{i}\left(-1\right)+\sum_{j=1}^{n_{2}}\mu_{j}=-1+\sum_{j=1}^{n_{2}}\mu_{j},$$
  which implies
  $$\sum_{j=1}^{n_{2}}\mu_{j}=1.$$
  Then,
  $$\sum_{j=1}^{n_{2}}\left(\mu_{j}+\delta_{j}\right)=1+\sum_{j=1}^{n_{2}}\delta_{j}=\sum_{j=1}^{n_{2}}w_{j}=1,$$
  and, consequently,
  $$\delta_{j}=0\;\text{and}\;\mu_{j}=w_{j}\text{,}\;\text{for}\;\text{all}\;j=1,\cdots ,n_{2}$$
  And, as in the first case,
  $$0_{p}=\sum_{i=1}^{n_{1}}\lambda_{i}x_{i}+\sum_{j=1}^{n_{2}}\mu_{j}\left(-y_{j}\right)=\bar{x}-\bar{y}.$$
(c) *b* = −1. Reasoning as in the case (b), we arise the same conclusion.

### Model definition for more than two data sets

In the case of more than two groups, we could proceed in two different ways:

1. Obtain the discriminant hyperplanes for each set with respect to the rest.
2. Obtain the discriminant hyperplanes that separate the given sets by pairs. In this case, if we have *k* different sets, we would obtain $\left(\begin{array}{c} k\\ 2 \end{array}\right)$ equations corresponding to the discriminant hyperplanes. For each group we will consider the subgroups of equations that separate it from the rest.

In **lpda** package we have implemented the second option.

### Overfitting problem

In nowadays it is very usual being involved in projects where the number of measured variables is much higher than the number of samples. In such cases, the high dimension allows statistical methods were succesfull separating groups. However, the hyperplane can overfit the training data and as a result a bad evaluation in the data test is obtained. To avoid this problem we propose obtaining the hyperplane from Principal Components (PCs) instead of the original variables. In general, when managing large amounts of noisy but correlated data, data analysis can greatly benefit from the application of dimensionality reduction methods, such as PCA, which allows the identification of the main patterns of variability avoiding residual or non-structural variation (examples in [14, 15]). Such approaches are effective in providing global understanding of most relevant information that can help to detect the differences between the studied groups.

PCA reduces the dimension of a set of individuals measured in a *p*-dimensional basis, taking advantage of the relationship between the variables. The method consists of projecting the individuals on a subspace of dimension *q* < *p* extracting the major information. The solution of this problem is the subspace defined by the *q* eigenvectors associated with the *q* higher eigenvalues of the variance-covariance matrix of the data. The selected number of PCs, *q*, is typically obtained on the basis of the percentage of the explained variability or by a cross-validation criterion. The PCA model corresponding to a data matrix **X**, of dimensions *n* × *p*, gives us the following decomposition:

$$\begin{array}{c} X=1_{n}\mu^{t}+TP^{t}+E \end{array}$$ (9)

where **1**_*n*_ is a size *n* column vector of ones, **μ**^*t*^ is a size *p* row vector containing estimates of de average for each variable, scores of the individuals in each PC are collected in the matrix **T**, the loadings (eigenvectors) are given by the matrix **P** and the residuals are collected in **E**.

The aplication of *LPDA* to the scores, or **T** matrix, will provide a classification hyperplane that avoids the undesirable noise focussing in the signal of interest. For more details about the PCA model and other projection techniques see [16].

## The evaluation strategy

To evaluate the performance of *LPDA* we first consider a data set with few variables to graphically inspect the behaviour of the hyperplane compared to *SVM*. Second, we consider an example of unbalanced and overlapping data between classes, with few variables but many individuals. Here the interest is to evaluate *LPDA* against other popular techniques such as *SVM*, *LDA* and Logistic Regression. Finally, we address a gene expression RNA-Seq data set, as example from the bioinformatics field, to show results with high-dimensional data. In this case, the method is compared with three classification techniques: *SVM* and two specific classification methods for RNA-Seq data. We describe the data and methods discussed below.

### Data sets

#### Palmdates

A data set with scores of 21 palm dates including their respective Raman spectra and the concentration of five compounds covering a wide range of concentrations: fibre, glucose, fructose, sorbitol and myo-inositol. The first 11 dates are Spanish (from Elche, Alicante) with no well-defined variety and the last 10 are from other countries and varieties, mainly Arabian. The data set is available in **lpda** package including two data.frames: conc with 5 variables and spectra with 2050. In this paper we use only conc data.

#### Default

A simulated data set containing information on 10.000 customers of which only 333 are default. It is an example of unbalanced data. The aim here is to predict which customers will default on their credit card debt, the minority class. This data set is in ISLR package [12].

#### Cervical cancer

A data set quantifying the expression of 714 microRNAs measured to 29 samples of tumor and 29 nontumor cervical tissue samples. This data set is available in Gene Expression Omnibus (GEO) Datasets with access number GSE20592 [17] and we normalized with *Quantile normalizaton* method described in [18].

### Classification methods

*SVM* is a hyperplane-based classification method, as said in the introduction. This method tries to find the hyperplane with the maximum margin that separates two classes, allowing some errors in the training set to avoid overfitting [4, 5]. Although *SVM* can also perform a non-linear classification, when dealing with so many variables there is no need of additional flexibility that will give polynomial or radial kernel models. For this reason, in next section we use linear classifiers, also called Support Vector Classifiers, for RNA-Seq example.

From the different packages avaible in **R** to apply *SVM* [19] we use the *SVM* implementation called **e1071**. The needed parameters in each application were computed with the crossvalidation proccess available in this package.

*Logistic Regression* considers a linear model where the response, a binary variable representing the class, is modelled with a logistic transformation. It is considered a specific case of Generalised Linear Models that are a generalization of classical Linear Models, which can accommodate a wider class of distributions named as exponential family, providing great flexibility for modeling different types of response variables. Normal, Poisson, Binomial and Gamma are examples of this family of distributions. In Logistic Regression, Binomial distribution is considered to model the response. More details in [3].

*LDA* computes the probabilities of belonging to each of the groups according to the available variables using Bayes Theorem (posteriori probability) and Normal distribution. The predicted class will be the one whose posteriori probability is maximum [1, 2].

Poisson Discriminant Analysis (*PDA*) [20] and Negative Binomial Discriminant Analysis (*NBDA*) [21] are specific methods for RNA-Seq samples classification. They can be considered as an extension of the *LDA* because they are Bayes rule-based classifiers taking into account the discrete count distribution inherent in these data.

## Results

We begin this section with palmdates data set to show a comparison between *LPDA* and *SVM* graphically. Then we show the results with Default data that is an unbalanced overlapped data set with a high number of samples where the separation is not possible. Finally, we present the application of *LPDA* and other methods to Cervical cancer RNA-Seq data.

### Palmdates data

**Fig 3:**
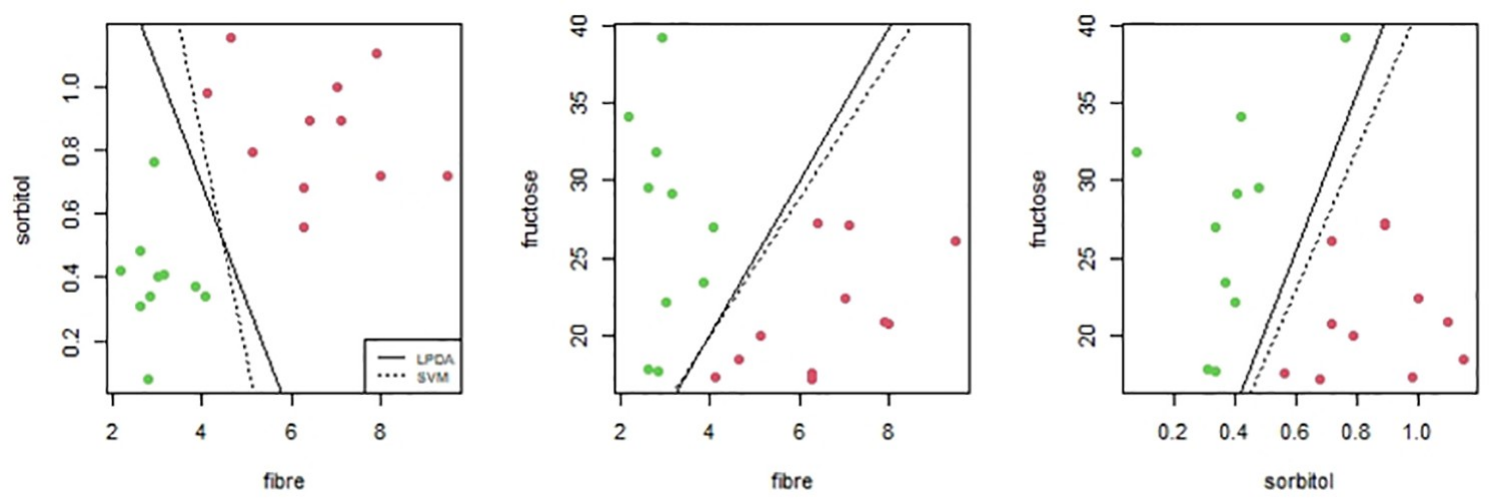
Examples where both methods are successfull but the hyperplanes are slight different.

**Fig 4:**
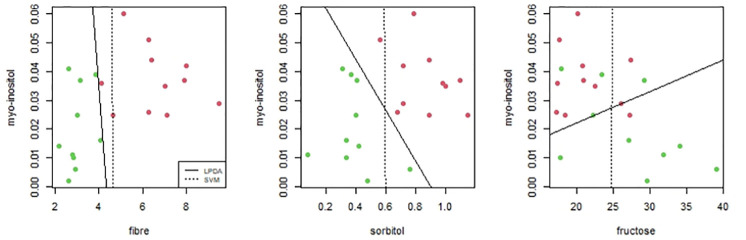
Examples where *LPDA* gets less separation errors than *SVM*.

As *SVM* and *LPDA* are methods based in hyperplanes separation, it is worth taking a closer look at this comparison. By comparing results of *SVM* and *LPDA* to different data sets we have seen that working with a high number of variables or having clear differences between groups, both methods are succesfull separating groups. However when having few variables or existing overlaps between groups, we find some differences. As example we show pairwise variables comparison of palmdates concentration data. We consider 4 variables: fibre, fructose, sorbitol and myo-inositol, avoiding glucose because it is highly correlated with fructose and gives repeated results. Fig 3 shows cases where both methods are successfull but the hyperplanes are slight different and Fig 4 shows cases where *LPDA* gets less separation errors than *SVM*: only one predicted error with *LPDA* in the third comparison meanwhile there are 1, 2 and 7 errors respectively with *SVM*.

### Default data

Another advantage we have found in *LPDA* with respect other techniques in several data sets is the good treatment of unbalanced data. These data is frequently encountered in biomedical and bioinformatics studies, where the group it is desired to predict is much smaller than the other one. General methods try to minimice the global error thus disadvantaging the minority class. Specific techniques are emerging for dealing with this problem [22]. Weights *m*_*i*_ and *w*_*j*_ considered by *LPDA* inside each group, mitigate this problem, meanwhile other techiques as *SVM* need to specify additional arguments when this situation arrises [23].

Default data is an example of unbalanced data that illustrates the problem clearly because only 0.3% of the data belongs to the group of interest (default class) that is desired to predict with low error. Table 1 shows sensitivity, especificity and the clasification error obtained with *LPDA*, weighted-*SVM*, Logistic Regression and *LDA*. We call weighted-*SVM* results of *SVM* applied considering as weights for each class the inverse of their sizes. As the interest is the good prediction in the default class, identified as the positive class, we must focuss in the sensitivity or percentage of True Positives detected. We observe that *LDA* and Logistic Regression give low sentitivity meanwhile *LPDA* gives a sensitivity very near the obtained with weighted-*SVM* and higher specificity, thus less global error.

**Table 1:** Sensitivity, specificity and classification error for default data.

	Sensitivity	Specificity	Classification error
*LPDA*	0.9009	0.8646	0.1342
weighted-*SVM*	0.9039	0.8555	0.1429
Logistic	0.3153	0.2372	0.0267
*LDA*	0.2372	0.9977	0.0276

### Cervical cancer RNAseq data

We applied *LPDA*, *SVM*, *POlda* and *NBlda*, to the cervical cancer data described before. Firstly, all the data was considered to compute the number of classification errors as a training set. None error was detected with *LPDA* and *SVM*. However, *POlda* and *NBlda* gave 3 and 4 classification error respectively, therefore, *LPDA* and *SVM* give a separate hiperplane meanwhile methods based in distributional assumptions do not.

We also evaluated the methods in test sets with a cross-validation strategy where the model was obtained 1000 times in different training and test sets. Table 2 shows the classification error rates average jointly to their confidence intervals. First, we notice the importance of the dimension reduction (*LPDA-PCA*) in this case, and in general when dealing with high dimensional data as RNA-Seq, which significantly reduces the error rate. We also observe that *LPDA-PCA* results are very similar to *SVM* and *NBlda* meanwhile *LPDA* without PCA results are similar to the *POlda* approach.

**Table 2:** Classification error test average and confidence interval in cervical cancer dataset.

Method	8 samples	10 samples	12 samples
*LPDA*	0.102 (0.096, 0.108)	0.106 (0.101, 0.112)	0.100 (0.095, 0.104)
*LPDA-PCA*	0.078 (0.072, 0.084)	0.078 (0.072, 0.083)	0.081 (0.076, 0.086)
*SVM*	0.076 (0.070, 0.082)	0.078 (0.073, 0.083)	0.081 (0.076, 0.085)
*POlda*	0.102 (0.096, 0.109)	0.105 (0.099, 0.110)	0.106 (0.101, 0.111)
*NBlda*	0.076 (0.071, 0.082)	0.082 (0.077, 0.087)	0.079 (0.075, 0.084)

## Conclusions

In this work, we propose a classification method based in a linear programming problem that is efficient in multiple scenarios. First, we show the basis of the method defining an optimization problem from the idea of separating two data sets in $R^{p}$. Then we consider the aplication of PCA when having overfitting problems due to high dimensional data and also usefull for correlated data. The method has been applied to different data sets and compared with popular techniques as *SVM*, Logistic Regression and *LDA*. One of these data sets is a real RNA-Seq data for which we considered the comparison with specific methods developed for the specific problematic of this type of data (*NBlda* and *POlda*).

Results show that *LPDA* is efficient in different situations. We have demonstrated its effectiveness in unbalanced experiments where it is able to classify minority classes without adding additional considerations. Moreover, its performance in high-dimensional data sets, such as RNA-Seq data, is similar to the popular *SVM* and also to *NBlda*, developed specially for the specific problematic of this type of data considering distributional hypothesis.

In this paper we have applied the method only in experiments where individuals are classified in two groups, but the method is extrapolated to three or more classes making pairwise comparisons in the available R-package.

In conclusion, *LPDA* is an efficient classification method for general multivariate data.
